# *Plasmodium vivax* inhibits erythroid cell growth through altered phosphorylation of the cytoskeletal protein ezrin

**DOI:** 10.1186/s12936-015-0648-9

**Published:** 2015-03-31

**Authors:** Tasanee Panichakul, Saranyoo Ponnikorn, Sittiruk Roytrakul, Atchara Paemanee, Suthathip Kittisenachai, Suradej Hongeng, Rachanee Udomsangpetch

**Affiliations:** Faculty of Science and Technology, Suan Dusit Rajabhat University, 204/3 Sirindhorn Rd. Bangplat, 10700 Bangkok, Thailand; Chulabhorn International College of Medicine, Thammasat University, 2nd Floor, Piyachart Building, Thammasat University, Rungsit campus, 12120 Patumthani, Thailand; Proteomics Research Laboratory, National Center for Genetic and Engineering and Biotechnology, National Science and Technology Development Agency, 113 Thailand Science Park, Phahonyothin Rd., Klong1, 12120 Klong Luang, Pathumthani Thailand; Department of Pediatrics, Faculty of Medicine Ramathibodi Hospital, Mahidol University, 272 Rama VI Rd., Ratchathewi District, 10400 Bangkok, Thailand; Department of Pathobiology, Faculty of Science, Mahidol University, 272 Rama VI Rd., Ratchathewi District, 10400 Bangkok, Thailand

**Keywords:** *Plasmodium vivax*, Ineffective erythropoiesis, Anaemia, Ezrin, Phosphoproteins, Haematopoiesis stem cells, Erythroid cells

## Abstract

**Background:**

The underlying causes of severe malarial anaemia are multifactorial. In previously reports*, Plasmodium vivax* was found to be able to directly inhibited erythroid cell proliferation and differentiation. The molecular mechanisms underlying the suppression of erythropoiesis by *P. vivax* are remarkably complex and remain unclear. In this study, a phosphoproteomic approach was performed to dissect the molecular mechanism of phosphoprotein regulation, which is involved in the inhibitory effect of parasites on erythroid cell development.

**Methods:**

This study describes the first comparative phosphoproteome analysis of growing erythroid cells (gECs), derived from human haematopoietic stem cells, exposed to lysates of infected erythrocytes (IE)/uninfected erythrocytes (UE) for 24, 48 and 72 h. This study utilized IMAC phosphoprotein isolation directly coupled with LC MS/MS analysis.

**Results:**

Lysed IE significantly inhibited gEC growth at 48 and 72 h and cell division resulting in the accumulation of cells in G0 phase. The relative levels of forty four phosphoproteins were determined from gECs exposed to IE/UE for 24-72 h and compared with the media control using the label-free quantitation technique. Interestingly, the levels of three phosphoproteins: ezrin, alpha actinin-1, and Rho kinase were significantly (p < 0.05) altered. These proteins display interactions and are involved in the regulation of the cellular cytoskeleton. Particularly affected was ezrin (phosphorylated at Thr567), which is normally localized to gEC cell extension peripheral processes. Following exposure to IE, for 48-72 h, the ezrin signal intensity was weak or absent. This result suggests that phospho-ezrin is important for actin cytoskeleton regulation during erythroid cell growth and division.

**Conclusions:**

These findings suggest that parasite proteins are able to inhibit erythroid cell growth by down-regulation of ezrin phosphorylation, leading to ineffective erythropoiesis ultimately resulting in severe malarial anaemia. A better understanding of the mechanisms of ineffective erythropoiesis may be beneficial in the development of therapeutic strategies to prevent severe malarial anaemia.

**Electronic supplementary material:**

The online version of this article (doi:10.1186/s12936-015-0648-9) contains supplementary material, which is available to authorized users.

## Background

*Plasmodium vivax* is a risk factor for severe anaemia, among patients in vivax-endemic areas [1-7]. Increasing evidence has established an association between vivax malaria, severe anemia, and death [8-16]. The pathogenesis of severe anaemia in vivax-malaria remains unclear and is likely caused by multiple underlying factors. These include the destruction of parasitized erythrocytes, ineffective erythropoiesis or dyserythropoiesis, and immunity associated with disease. Evidence for dyserythropoiesis, pancytopenia and degradation of erythroblasts was found in bone marrow from patients infected with *P. vivax* parasites [17-21]. Moreover, *in vitro* cultures of erythroid cells derived from haematopoietic stem cells has demonstrated that *P. vivax* is able to directly inhibit erythroid cell proliferation and differentiation [22]. The molecular mechanisms underlying the suppression of erythropoiesis by *P. vivax* are remarkably complex and poorly understood.

The phosphoproteome strategy is alternative proteomic method that allows investigation into the molecular mechanisms of signal transduction pathways [23]. The separation and enrichment of phosphoproteins utilizes metal ion or TiO_2_ embedded columns prior to the identification and determination of phosphoproteins under liquid chromatography–mass spectrometry (LC-MS) based techniques [24,25]. Many molecular pathways in eukaryotic cells are modulated by specific signaling proteins that are controlled, by phosphorylation and dephosphorylation, through the activity of kinase and phosphatase enzymes. This post-translational control of eukaryotic cellular machinery is a hallmark of pathways that respond to different stimuli. The level of protein phosphorylation at specific sites varies from less than 1% to greater than 90%, depending on conditions [26]. The regulation of complex and dynamic signal transduction proteins contributes to the destination of targeting proteins and the signal transduction of cell growth, and exposure to parasites can also influence signaling pathways. This occurs through specific modulation of regulatory proteins during the host-pathogen interaction, especially proteins with roles in pathogenesis [27]. The specific mechanism involved in the suppression of erythroid development by *P. vivax* has not been elucidated. However, it is known that during parasite exposure, suppressed erythroid development is a key aspect in the pathophysiology of anaemia. Here, this study describes the first comparative phosphoproteome of erythroid cells, derived from human haematopoietic stem cells, exposed to proteins of *P. vivax*. This analysis utilized IMAC phosphoprotein isolation, directly coupled with LC MS/MS analysis. Interestingly, the phosphoprotein ezrin, involved in the regulation of cytoskeleton during cell development, was identified using this procedure. Ezrin, a member of the ezrin, radixin, and moesin (ERM) subfamily of cytoskeletal proteins and is conserved both functionally and structurally in mammalian cells [28]. Through the regulation of the cytoskeleton ezrin has a role in several cellular processes, such as maintenance of survival, cytokinesis, adhesion, membrane dynamics, motility, and integration of membrane transport with signaling pathways [29]. Usually, ezrin is present in an inactive conformation and functions as a crosslinker between the plasma membrane and cytoskeleton [30]. Two regions of ezrin, the N-terminal and C-terminal domains are involved in the active conformation. The N-terminal domain binds to phosphatidylinositol 4,5-biphosphate, which facilitates phosphorylations of threonine 567 (Thr567) in the C-terminal domain [28]. Rho kinase and protein kinase C (PKC) are capable of phosphorylating the C-terminal threonine in ezrin, promoting the formation of the F-actin binding site in active conformation [31-35]. This study has identified the involvement of the cytoskeleton protein ezrin in the inhibitory effect of *P. vivax* on erythroid cell growth, leading to ineffective erythropoiesis. The molecular mechanism characterized in this study, relevant to ineffective erythropoiesis, should have utility in the development of therapeutic strategies for severe malarial anaemia.

## Methods

### Parasite preparation

*Plasmodium vivax* parasites from patient blood with 0.05-0.2% parasitaemia, as determined by examining thick and thin blood smears, were collected. The ethical and methodological aspects of this study for parasite collection from patients attending the malaria clinic in Tha Sae, Chumpon Province, Thailand (MU-IRB 2012/170.2511) have been approved by the Mahidol University Institutional Review Board, Mahidol University, Bangkok, Thailand. Infected erythrocytes (IE) were separated from patient blood using a 60% Percoll solution as previously described [36]. Briefly, 20 ml of whole blood from patients were collected and passed through a Plasmodipur filter (Euro-Diagnostic B.V., Netherlands) to remove white blood cells. To separate IE, patient blood, after filtration, was diluted 1:20 with RPMI1640 (Invitrogen®, CA, USA), layered on 60% Percoll, and centrifuged at 1,200 g for 20 min at 20°C. The purity of IE after isolation was 95%, containing 80% schizonts and 20% other stages. The isolated IEs were used as lysed cells prepared by freezing and thawing as previously described [22].

### Isolation of cord blood CD 34^+^ cells and culture conditions

Haematopoietic stem cells (HSCs)/CD 34^+^ cells were isolated from normal human umbilical cord blood as previously described [22]. Cord blood collected from normal full-term deliveries at Ramathibodi Hospital (ID 04-52-39) was approved by the Ethical Committee of Research on Human Beings of the Ramathibodi Hospital, Faculty of Medicine, Mahidol University. Briefly, HSCs/CD34^+^ cells from cord blood were isolated using a CD 34 isolation kit with magnetic microbead Mini-MACS columns (Miltenyi Biotech, Geramany). The purity of CD34^+^ cells after isolation was 97% as determined by flow cytometry analysis.

Growing erythroid cells (gECs), derived from HSCs/CD34^+^ cells, at a density of 5 x 10^5^ cell/well in 24-well tissue culture plates (Corning Incorporated Costar®, NY, USA) were cultured in 1 ml of complete medium containing Stemline II medium (Sigma-Aldrich Corporation, Missouri, USA) supplemented with cytokines as previously described [36]. Lysates of IE or uninfected erythrocytes (UE) from normal donor blood were added to gEC cultures, at a ratio of 1:10 (gEC:IE/UE), on day 5 and cultured at 37°C in 5% CO_2_ for 24, 48, 72 h. Viable cells were determined by trypan blue dye exclusion and dead cells were stained with 2 μg/ml propidium iodide (eBioscience) and analysed by flow cytometry.

### Cell cycle analysis

gECs exposed to IE/UE for 48 and 72 h were washed with PBS and fixed with 70% cold ethanol overnight at -20°C. After fixed cells were washed with PBS, cells were added with 5 μg/ml RNase A (Geneaid Biotech Ltd., Taiwan), stained with 20 μg/ml propidium iodide (eBioscience) and incubated for 30 min at 37°C in dark. DNA analysis was performed on a FACS Canto™ flow cytometer (Becton Dickinson). Cell cycle analysed by BD FACSDiva software version 4.1 (Becton Dickinson) was used to determine the percentages of cells in the different cell cycle phases. Experiments were performed five independent times and values are shown as means ± S.D.

### Preparation of phosphoprotiens

Phosphoproteins were prepared from gECs, after exposure to IE/UE lysates, using Pierce Phosphoprotein enrichment kit (Thermo Scientific, Pierce Biotechnology, IL, USA) followed the manufacturer’s instructions. Briefly, 5 x 10^6^ cells were added to 200 μl of lysis/binding/wash buffer with CHAPS and 1X phosphatase inhibitors (phosphatase inhibitor cocktail 100X, Roche, UK) and lysed on ice by sonication (Sonics Vibra cell,sonics & materials INC., USA) with amplitude 70% utilizing 15 cycles of 2 sec disruption followed by 2 sec on ice. Following centrifugation (Tomy MX-305 high speed refrigerated microcentrifuge, CA, USA), 10,000 g at 4°C for 10 min, supernatants from cell lysates were collected and proteins concentrations were determined by Lowry method [37].

For phosphoprotein enrichment, cell lysates were adjusted to a concentration to 0.5 mg/ml in lysis/binding/wash buffer without CHAPS and applied to the column. Columns were inverted to mix for 30 min at 4°C and then washed three times with lysis/binding/wash buffer with CHAPS using centrifugation (Allegra X-22R centrifuge, Beckman Coulter, Inc, USA) at 1,000 g for 1 min at 4°C. One millimetre of elution buffer, consisting of 75 mM sodium phosphate, 500 mM sodium chloride, pH 7.5, was added to each column and then incubated at room temperature, with agitation for 3 min. The eluted proteins were collected by centrifugation at 1,000 g for 1 min at 4°C, and this step was repeated four times. The pool of eluted proteins was concentrated using a Pierce concentrator (Thermo Scientific). Briefly, 4 ml of each sample of eluted proteins was placed into the upper chamber of the concentrator followed by centrifugation at 7,000 g for 30 min at 4°C. The concentrated proteins, approximately 100-200 μl, were collected from the upper chamber and stored at -80°C. In the final step, salts and other molecules (<1,000 Da) were removed using Zeba™ spin desalting columns (Thermo Scientific). One hundred microlitres of each sample was applied to columns and then centrifuged at 700 g for 30 sec. The desalted samples were collected and the proteins content of each sample was determined by the Lowry method and used for phosphoprotein analysis.

### Phosphoprotein analysis

After desalting, 4 μg of phosphoproteins in 10 mM ammonium bicarbonate were reduced with 10 mM DTT (Dithiotheitol) for 30 min at 60°C, alkylated with 15 mM IAA (iodoacetamide) at room temperature for 30 min, and digested with sequencing grade trypsin (Promega, Geramany) for 16 h at 37°C. Tryptic phosphopeptides were diluted with 0.1% formic acid to final concentration of 0.25 μg/μl and centrifuged 10,000 rpm at room temperature for 10 min. Phosphopeptide samples were injected into a NanoAcquity system (Waters Corp., Milford, MA) equipped with a Symmetry C_18_ 5 μm, 180-μm x 20-mm Trap column and a BEH130 C_18_ 1.7 μm, 100-μm x 100-mm analytical reversed phase column (Waters Corp., Milford, MA). The samples were initially transferred with an aqueous 0.1% formic acid solution (mobile phase A) to the trap column with a flow rate of 15 μl/min for 1 min. The peptides were separated with a gradient of 15-50% mobile phase B in acetonitrile with 0.1% formic acid over 15 min at a flow rate of 600 nl/min followed by a 3 min rinse with 80% of mobile phase B. The column temperature was maintained at 35°C. The lock mass was delivered from the auxiliary pump of the NanoAcquity system with a constant flow rate of 500 nl/min with 200 fmol/μl of [Glu^1^] fibrinopeptide B delivered to the reference sprayer of the NanoLockSpray source of the mass spectrometer. All samples of tryptic peptides were analysed by using a SYNAPT™ HDMS mass spectrometer (Waters Corp., Manchester, UK), which was operated in the V-mode of analysis with a resolution of at least 10,000 full-width half-maximum, using positive nanoelectrospray ion mode. The time-of-flight analyzer of the mass spectrometer was externally calibrated with [Glu^1^] fibrinopeptide B from m/z 50 to 1600 with acquisition lock mass corrected using the monoisotopic mass of the doubly charged precursor of [Glu^1^] fibrinopeptide B. The reference sprayer was sampled with every 20 sec. Accurate mass LC-MS data were acquired with data direct acquisition mode. The energy trap was set at the collision energy of 6 V. In the transfer collision energy control, low energy was set at 4 V. The quadrupole mass analyzer was adjusted such that ions from m/z 300 to 1800 were efficiently transmitted. The MS\MS survey was over range from 50 to 1990 Da and scan time was 0.5 sec.

### Protein identification

The differential quantitation of proteins and peptides based on the MS signal intensities of individual LC-MS samples was analysed using DeCyder MS differential analysis software (DeCyderMS, GE Healthcare) [38,39]. Acquired LC-MS raw data were converted and the PepDetect module was used for automated peptide detection, charge state assignments, and quantitation based on the peptide ions signal intensities in MS mode. The analysed MS/MS data from DeCyderMS were submitted for database search using the Mascot software (Matrix Science, London, UK) [40]. For protein identification, data was compared against the NCBI *Homo sapiens* database with the following parameters: enzyme (trypsin); variable modifications (carbamidomethyl, oxidation of methionine residues); mass values (monoisotopic); protein mass (unrestricted); peptide mass tolerance (1.2 Da); fragment mass tolerance (±0.6 Da), peptide charge state (1+, 2+ and 3+) and max missed cleavages.

### Bioinformatic analysis

All MS/MS data were compared against the human protein set using Swissprot protein database. The heatmap visualization was constructed using the web-based analysis MEV program [41] and the protein cluster was analysed by distance metric selection with Pearson correlation parameters calculate K-Means. Different levels of phosphoproteins were analysed using the significant *t*-test at *P*-value ≤ 0.05. The gene ontology analysis was performed with UniprotKB (protein knowledgebase) [42] and PANTHER (classification system) [43] databases for biological processes, molecular function and classification. The mapping of protein-protein interactions was conducted using data visualization with statistical analysis at a low confidence score in the STRING database (known and predicted protein-protein interactions) [44]. Gene biological categorization was performed and selected at *P*-value ≤ 0.01. Analysis of the protein-protein interaction network was performed using the KEGG (Kyoto Encyclopedia of Genes and Genomes) PATHWAY database [45,46].

### Immunofluorescence assays

The distribution of threonine-phosphorylated ezrin and ezrin in gECs after exposure to IE/UE and in control media was verified by immunofluorescence. Cells were fixed in 4% formaldehyde in PBS (phosphate buffer saline) at 4°C for 20 min, washed with PBS, permeabilized with 0.1% Triton X-100 in PBS for 10 min, and blocked in 2% human serum in PBS for 20 min at room temperature. Mouse anti-ezrin (Abcam®, Cambridge, UK) and rabbit anti-phospho-ezrin (Thr567)/radixin (Thr564)/mosin (Thr41A3) ERM (Cell Signaling Technology, Danvers, MA, USA) antibodies, diluted in 1% human serum/PBS at a ratio 1:100, were added and incubated at room temperature for 1.5 h. Cells were incubated with goat anti-rabbit and anti-mouse antibodies, conjugated with Alexa Fluor green 488 and Alexa Fluor Red 594 (Molecular probes), respectively, diluted in 2% human serum/PBS were for 2 h. After washing with PBS, stained cells were mounted with anti-fade medium containing DAPI (Molecular probes). Cells were examined using a laser scanning confocal microscope (LSM 510 Meta, Zeiss, Jena, Germany) with a 63x objective at zoom 2. Immunofluoresence intensity of 1,000 cells from each culture condition was determined using ImageJ 1.48v/Java software [47]. The mean of intensity from 1,000 cells were presented as intensity ratios, calculated from the intensity of IE/UE-exposed gECs divided by the intensity of gECs in control cultures.

### Statistical evaluation

Data for cell growth was analysed using the SPSS program (version 17). The unpaired Mann-Whitney-Wilcoxon test was used to compare means between independent groups for significantly statistic evaluation of cell growth, *P*-value < 0.01.

## Results

### Inhibition of erythroid cell growth in *in vitro* cultures by *P. vivax*

**Figure 1:**
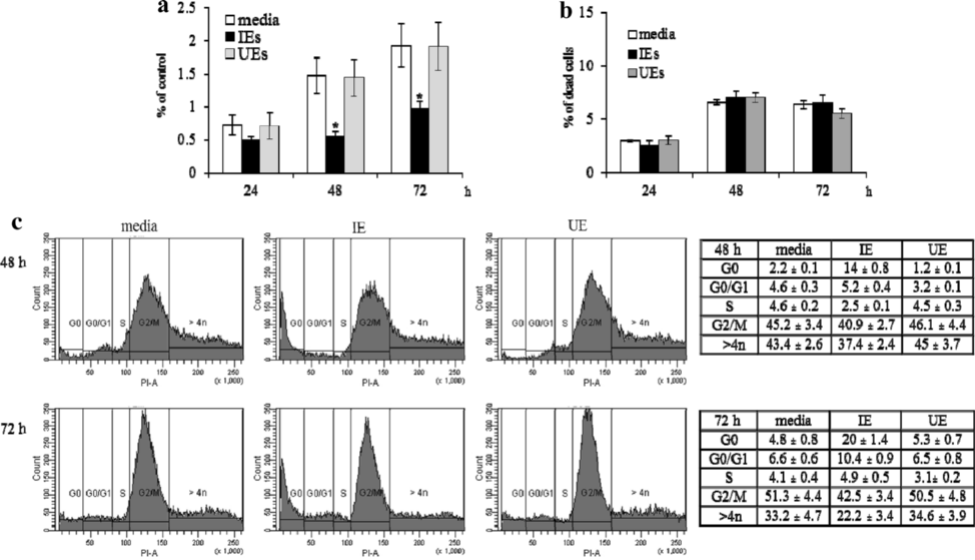
**Inhibitory effect of** ***P. vivax*** **on erythroid cell growth and cell cycle.** gECs, 5-day old, were cultured with IE/UE lysate at a ratio of 1:10 (gEC:IE/UE) for 24, 48 and 72 h. **(a)** Cell growth is presented as means ± SD of percentage of control, compared with a control containing medium. **(b)** Cell death is illustrated as means ± SD of percentage of dead cells, and **(c)** cell cycle is illustrated as means ± SD of percentage of DNA content in each phase of G0, G0/G1, S, G2/M and > 4n, using propidium iodide staining. Means ± SD were calculated from five independent experiments. *P-value < 0.01 compared with media control.

It was previously reported that *P. vivax* parasites inhibited erythroid cell growth and perturbed erythroid cell division in three-day *in vitro* cultures [22]. However, the molecular mechanisms underlying the inhibition of erythroid development by *P. vivax* were unclear. In this study, the underlining mechanism of the inhibitory effect on gEC growth by *P.vivax* was examined using growing erythroid cells (gECs), derived from human cord blood HSCs/CD34^+^, and infected erythrocytes (IE), isolated from patient blood. gECs from 5-day old cultures were exposed for 24, 48 and 72 h to lysed IE or UE, at a ratio of IE/UE: gEC 10:1. Lysed IE significantly inhibited gEC growth at 48 and 72 h (*P*-value < 0.01), compared with lysed UE and media controls (Figure 1a). No difference in cell death at 24, 48 and 72 h was observed in cultures with and without IE/UE, as shown in Figure 1b. Interestingly, lysed IE dramatically increased the number of cells in the G0 phase and decreased the number of cells in the mitotic fraction (G2/M and > 4n) at 48 and 72 h (Figure 1c). This indicates that parasite proteins were able to inhibit erythroid cell growth and division resulting in the accumulation of cells in a resting phase but did not induce cell death.

### Characterization of phosphoproteins from erythroid cells after exposure to *P. vivax*

**Figure 2:**
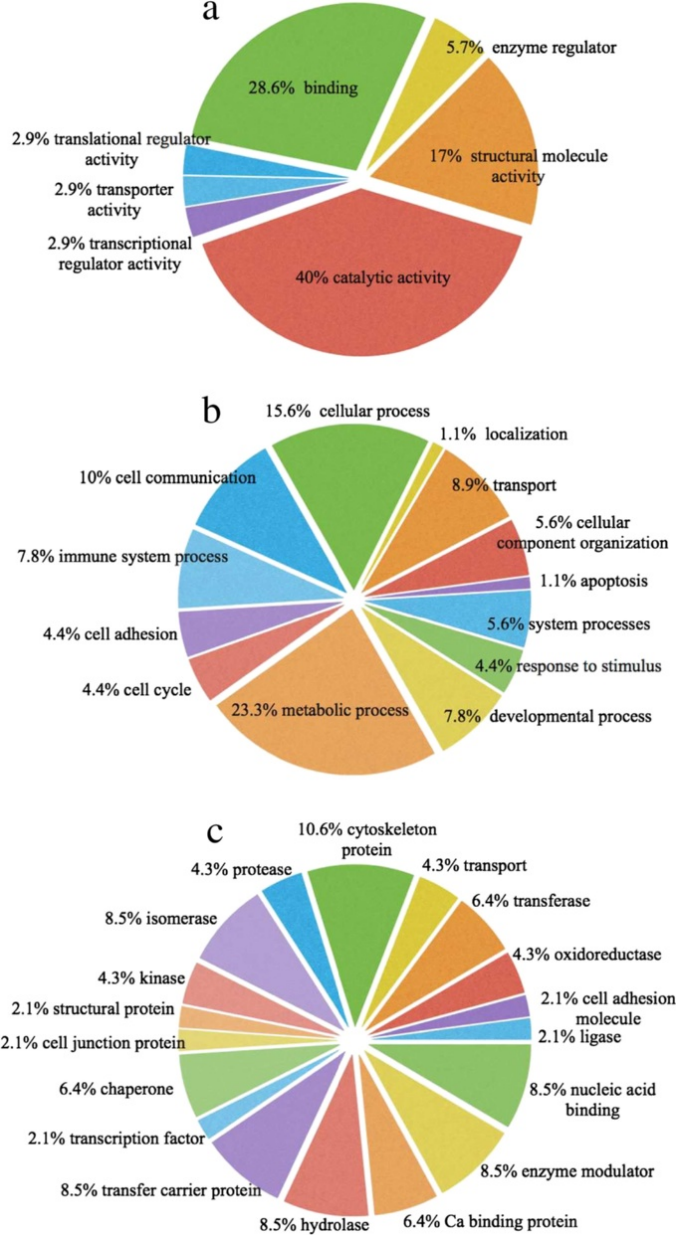
**Classification of phosphoproteins identified in erythroid cells.** Phosphoproteins found following exposure to infected erythrocytes (IE)/uninfected erythrocytes (UE) and in media control were categorized according to **(a)** molecular functions, **(b)** biological processes, and **(c)** protein classes.

To analyse the mechanism of *P. vivax* inhibition on gEC growth, following exposure to IE/UE for 24, 48 and 72 h, phosphoproteins were enriched and analysed by LC-MS/MS. The DecyderMS program module was used for quantitation of the MS/MS intensity in each culture condition and results were compared to the human protein database. Phosphoproteins were classified based on biological function, molecular function, and protein class. Forty four phosphoproteins were identified from gECs exposed to IE/UE and media control at 24, 48 and 72 h (Figure 2). The molecular functions of the 44 phosphoproteins were classified according to the gene ontology analysis (Figure 2a) and include those involved in catalysis (40%), binding (28.6%), structural molecules (17%), enzyme regulators (5.7%), translation regulators (2.9%), transcription regulators (2.9%) and transporters (2.9%). The biological processes of these phosphoproteins (Figure 2b) were found to be predominantly in metabolic processes (23.3%), cellular processes (15.6%), transport (8.9%), and cell cycle (4.4%). In addition, protein class categories were analysed for these phosphoproteins indicating functions in cytoskeleton (10.6%), proteases (4.3%), kinases (4.3%) and structural proteins (2.1%) (Figure 2c).

**Figure 3:**
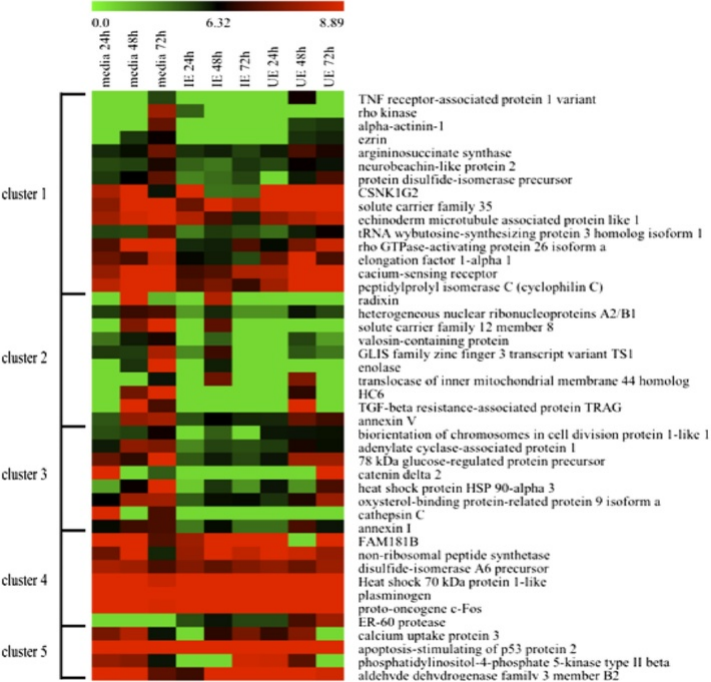
**Comparison of phosphoprotein levels in erythroid cells untreated and exposed to** ***P. vivax*** **.** Heatmap of relatively high (red) and low (green) phosphoproteins from gECs exposed to lysates of infected erythrocytes (IE) and uninfected erythrocytes (UE) for 24-72 h and gECs in media control. The heatmap and clustering were generated using the web-based analysis program MEV.

Forty four phosphoproteins, identified from gECs exposed to IE/UE for 24 – 72 h, are presented using a heatmap visualization (Figure 3). The relative levels of these phosphoproteins was evaluated as high and low compared with level of phosphoprotein present in gECs from media control. The patterns of phosphoproteins from gECs exposed to IE/UE or in media for 24, 48 and 72 h were analysed in five clusters and categorized using the K-mean clustering method (Figure 3). Interestingly, the relative level pattern of phosphoproteins in cluster 1 was low from gECs exposed to IE for 24 – 72 h. To identify potential phosphoproteins from five clusters involved in inhibition of gEC growth and division by IE, the relative levels of phosphoproteins in each condition and time point were determined using statistical analysis with paired *t*-test at *P*-value ≤ 0.05. The lists of selected phosphoproteins with significant differences in relative level (*P*-value ≤ 0.05) were evaluated for biological processes and molecular functions using the KEGG PATHWAY database, as shown in Table 1. Interestingly, three of the phosphoproteins identified, ezrin, alpha actinin-1 and Rho kinase were reported to function in the regulation of the cellular cytoskeleton (Table 1). Ezrin, alpha actinin-1 and Rho kinase from gECs were observed to be all included in cluster 1 and the relative levels of these three phosphoproteins were significantly low from gECs exposed to IE for 72 h, compared with gECs in media only. These findings suggests that ezrin, alpha actinin-1 and rho kinase in the phosphoproteome of gECs may have roles relevant to the cellular pathology caused by *P. vivax* during erythroid cell growth.

**Table 1:** **Evaluation of biological processes and molecular function of selected phosphoproteins using the KEGG PATHWAY database**

**Cluster**	**KEGG pathway**
	**Protein processing in endoplasmic reticulum** ^**a**^
2	Valosin-containing protein
4	ER-60 protease^b^
4	Heat shock 70 kDa protein 1-like
4	Disulfide-isomerase A6 precursor
	**Regulation of actin cytoskeleton** ^**a**^
1	Rho kinase
5	Phosphatidylinositol-4-phosphate 5-kinase type II beta
1	Ezrin^b^
1	alpha actinin-1^b^
2	Radixin
	**Leukocyte transendothelial migration** ^**a**^
1	Rho kinase
1	Ezrin^b^
1	alpha actinin-1^b^
	**Focal adhesion**
1	Rho kinase
1	alpha actinin-1^b^
	**MAPK signaling pathway**
4	Heat shock 70 kDa protein 1-like

^a^KEGG pathway analysis under STRING database at p value ≤ 0.01.

^b^The differential relative level of phosphoproteins under significant *t*-test at p value ≤ 0.05.

**Figure 4:**
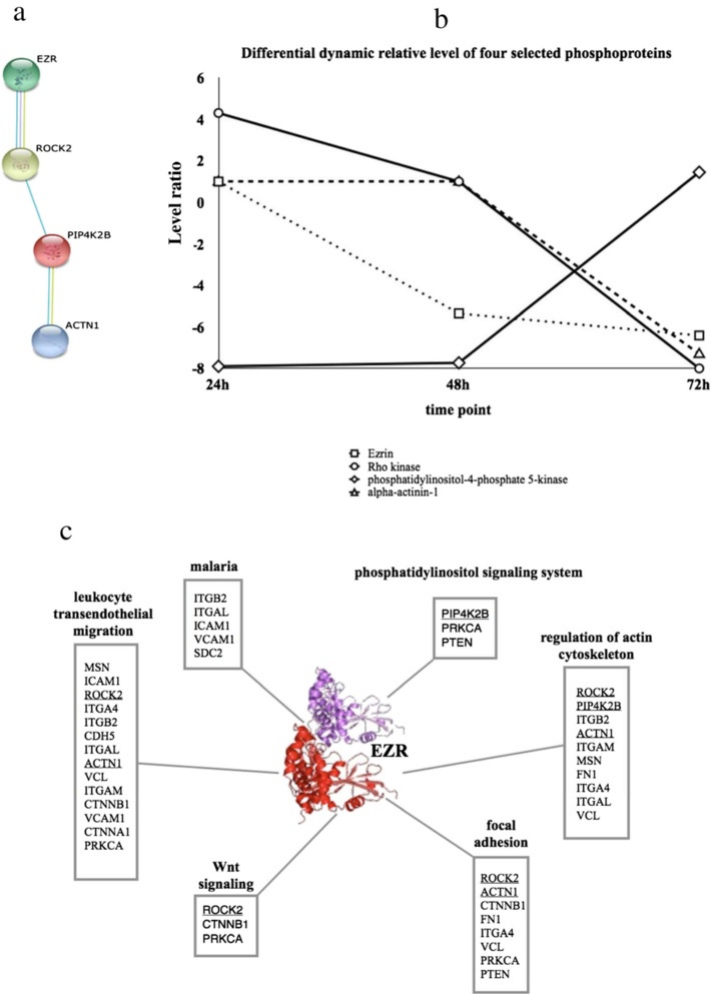
**Relative levels and interactions of selected phosphoproteins. (a)** Interactions of ezrin, Rho kinase, phosphatidylinositol-4-phosphate 5-kinase type II beta and alpha actinin-1 analysed using the STRING database **(b)** relative levels of selected phosphoproteins, ezrin, Rho kinase, phosphatidylinositol-4-phosphate 5-kinase type II beta and alpha actinin-1, and **(c)** interaction of ezrin with regulatory pathways and cellular cytoskeleton generated using the KEGG PATHWAY database.

Exposure to a pathogen, like parasites, has the ability to disturb the cellular processes in particular cell types. The alteration of protein expression and function inside the cells are the result of host-pathogen interactions. The exploring protein-protein interactions of gECs exposed to IE lysate were analysed using web based free database, STRING and three significant relative levels of phosphoproteins including ezrin, alpha actinin-1and Rho kinase were specifically found in the networks (Figure 4a). Moreover, the finding of phosphatidylinositol-4-phosphate 5-kinase type II beta in cluster 5 (Figure 3) also links the association of three proteins (Figure 4a). Interestingly, the protein-protein interaction of ezrin, alpha actinin-1, Rho kinase and phosphatidylinositol-4-phosphate 5-kinase type II beta was found in a specific relevant interaction pathway of IE-exposed gEC. Likewise, the alteration of defined phosphoprotein levels in gECs during exposure to IE for 24 to 72 h was performed as the dynamic relative level of phosphoproteins as shown in Figure 4b. Levels of the phosphoproteins ezrin, alpha actinin-1 and rho kinase are reduced following 48 to 72 h of IE exposure to gECs, compared with media controls. In contrast, abundance of phosphatidylinositol-4-phosphate 5-kinase type II beta was elevated under the same conditions. This showed that the altered relative level of these 4 phosphoproteins was a result of the interactions between IE and gEC proteins. To investigate whether reduced abundance of ezrin is in-volved in the mechanism of ineffective erythropoiesis in malaria, the ezrin protein-protein interaction network was analysed using the KEGG PATHWAY database. The ezrin interactome and its involvement in various cellular functions is shown in Figure 4c. This analysis revealed that the ezrin protein has important roles in various pathways though its function in the regulation of actin cytoskeleton may be the most relevant to malaria. While ezrin is associated with several pathways, only regulation of the actin cytoskeleton is shared among alpha actinin-1, Rho kinase and phosphatidylinositol-4-phosphate 5-kinase type II beta (Figure 4c).

### Inhibitory effect on erythroid cell growth by *P. vivax* is regulated by ezrin phosphorylation

**Figure 5:**
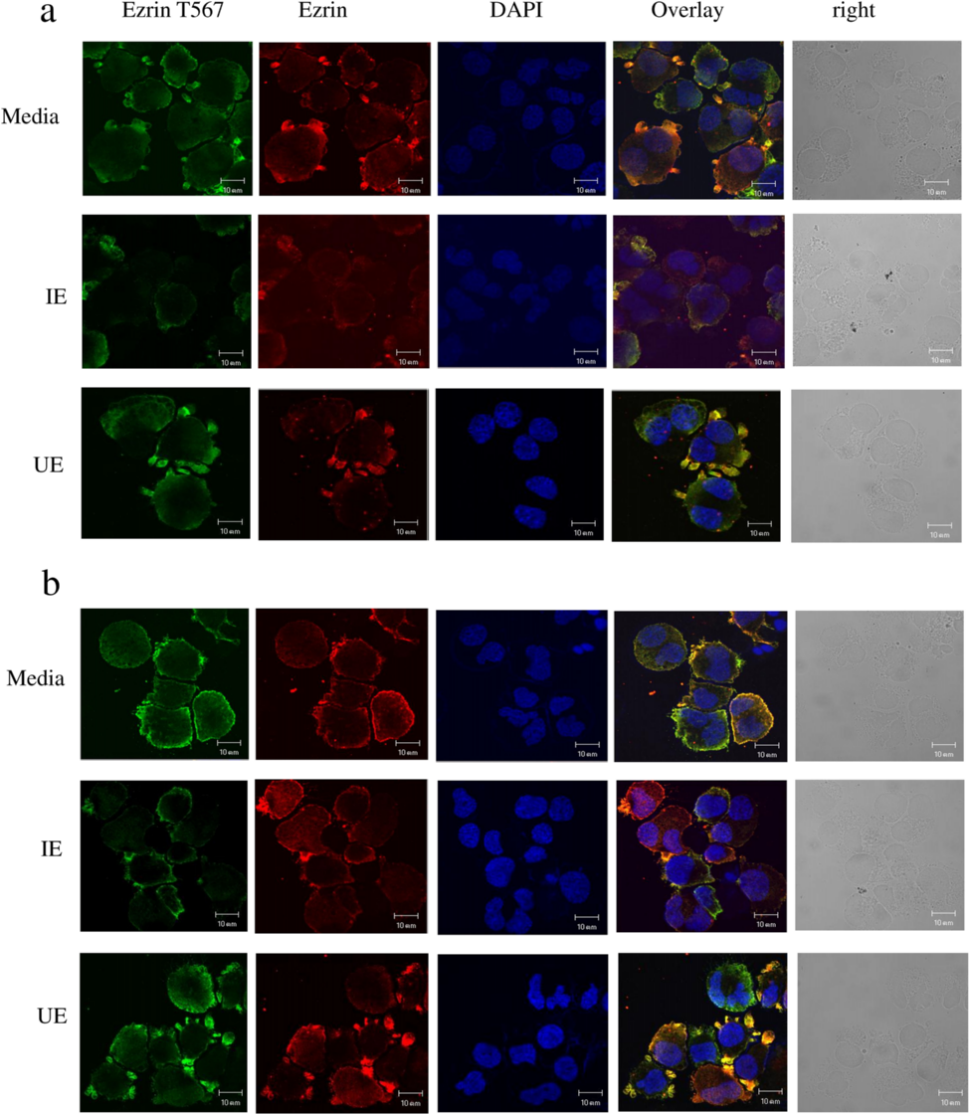
**Localization of ezrin and phospho-ezrin Thr 567 in untreated erythroid cells and those exposed to** ***P. vivax*** **.** Immunofluorescence, ezrin phospho-Thr567 (green), ezrin (red), and nucleus (blue) from gECs exposed to lysates of IE/UE and cells in media for 48 h **(a)** and 72 h **(b)** detected with using specific antibodies against ezrin and phospho-ezrin Thr567. Images were captured using a confocal microscope with 63x magnification and zoom 2.

Protein phosphorylation is involved in several regulatory functions in living cells. Using phosphoproteomic and bioinformatics analysis, ezrin was identified and determined to have to function in regulation of actin cytoskeleton. One ezrin phosphopeptide from gECs contained the amino acid sequences DK**Y**K**T**LRQIR, corresponding to residues 563-572. The phosphorylation site of this peptide analysed from MS/MS data was predicted to be tyrosine 565 (Y565). Ezrin, and phospho-ezrin were evaluated by immunofluorescence using specific antibodies against ezrin and phospho-ezrin Thr567 [31], as shown in Figure 5. Unfortunately, the antibody against phospho-ezrin Y565 is not available and data for phosphorylation at this ezrin residue was not reported. Results of immunofluorescence showed that ezrin and phospho-ezrin Thr567 localized to cell extensions peripheral processes of gECs. gECs in culture with UE or in media displayed strong signals for phospho-ezrin Thr567. In contrast, the signal strength for phospho-ezrin Thr567 was markedly reduced following exposure to IE for 48 and 72 h (Figure 5a and b). Quantitation of signals using ImageJ software for ezrin and phospho-ezrin Thr567 from 1,000 gECs from each culture, confirm the decreased level of ezrin proteins in cells exposed to IE (Additional file 1). The intensity ratios of phospho-ezrin Thr567 in IE-exposed gECs compared to media controls were less than 1 (0.17 and 0.26, at exposure times 48 and 72 h, respectively). In contrast, the same analysis using UE-exposed gECs gave intensity ratios for phospho-ezrin Thr567 of approximately 1 (0.99 and 0.93, at exposure time 48 and 72 h, respectively). Ezrin signals in IE/UE-exposed gECs compared to gECs in media at 48 and 72 h were nearly 1 (0.86 and 0.95 for IE-exposed gECs, and 0.9 and 0.98 for UE-exposed gECs, respectively), as shown in Additional file 1. These results indicate that ezrin phosphorylation at the carboxy-terminal threonine residue 567 was decreased in gECs exposed to IE. This suggests that parasite proteins were able to inhibit erythroid cell growth by preventing the phosphorylation of the C-terminal region of ezrin.

## Discussion

Anaemia has been frequently observed to be a consequence of vivax infection [4,7,10,48-58]. Recently, transmission electron microscope analysis of two vivax malarial cases with symptom of anaemia demonstrated the presence of *P. vivax*-infected erythroblasts and subsequent degradation of erythroblasts. Interestingly, *P. vivax* parasites were found in bone marrow, but could not be detected in peripheral blood [17]. Consistent with this observation, an *in vitro* study has revealed that *P. vivax* can directly inhibit erythroid cell proliferation and differentiation through altered division of erythroid cells [22]. However, the mechanisms underlying the suppression of erythroid development by *P. vivax* appear to be complex and poorly characterized. In this study, phosphoprotein analysis was used to investigate the underlying the suppression of erythroid development by *P. vivax*. Using LC-MS/MS analysis in combination with gene ontology information from the human protein database, 44 phosphoproteins from gECs exposed and non-exposed to IE/UE were identified and categorized according to molecular function, biological process and protein class. Interestingly, the relative level of phosphoproteins was significantly lower following exposure of gECs to parasite proteins, compared to non-exposed cells. Phosphoproteins that displayed significant decreases include ezrin, alpha actinin-1 and Rho kinase. These phosphoproteins were determined to be in the same cluster 1 with significantly low level pattern of phosphoproteins and exhibited a similar pattern of abundance in response to IE exposure. These results suggest that the phosphoproteins ezrin, alpha actinin-1 and Rho kinase may have roles in the inhibitory effect on erythroid cell growth and division following *P. vivax* exposure. The protein-protein interaction networks determined using the KEGG human protein database revealed that ezrin functions in several cellular pathways, including regulation of actin cytoskeleton.

Ezrin, a member of ERM (ezrin, radixin and moesin) proteins, is localized to cell extension peripheral processes and able to interact with transmembrane proteins and the cytoskeleton. Ezrin functions to regulate cytoskeletal assembly, membrane-protein function and membrane transport [29,59,60]. Recent studies, have implicated ezrin as a signal transducer involved in a wide variety of cellular functions, including cell survival, adhesion, morphogenesis, motility, cytokinesis and cellular proliferation [61,62]. Increasing evidence indicates a direct relationship between cell proliferation and the level of ezrin expression in cells [63-65]. Microinjection of anti-ezrin antibodies into the cytoplasm blocked the entry of mouse fibroblasts into S phase, confirming the function of ezrin in proliferation [66]. In response to TNF, ezrin inhibits endothelial cell proliferation through transcriptional repression of cyclin A, a cell cycle regulatory protein [67]. This study also found that ezrin in erythroid cells appears to function in the regulation of cell growth and division. Erythroid cells with growth inhibited by *P. vivax* display low levels of ezrin phosphoprotein.

The phosphorylation of a threonine at residue 567 (T567) is necessary for the active conformation of ezrin and enhances binding with F-actin [28]. The results of this study also find that phosphorylation of ezrin, possibly at Thr567, is involved in the regulation of erythroid cell growth. Although our analysis identified the position of the phosphorylation site in an ezrin-phosphopeptide as tyrosine 565, the phosphorylation of ezrin threonine 567 was also detected using a specific antibody. A source of error in phosphorylation site localization on peptides is the presence and intensity of ions for these species in the MS/MS spectra [68]. However, both residues 565 and 567 are contained in the phosphopeptide 563-572 from ezrin, which was detected in this study. It is also possible that the anti-ezrin Thr567 monoclonal antibody may recognize the phosphorylation of Tyr565 as well as Thr567. This antibody was produced using phosphopeptide of human ezrin containing residues 564-568 (K**Y**K**T**L) [31] and contains both Tyr565 and Thr567. Previous reports have demonstrated the specificity of this antibody for phospho-ezrin Thr567 [69-71] but it is not known if it can recognize phosphorylation at other sites.

Three phosphoproteins in this study, ezrin, Rho kinase and alpha actinin-1 are present at significantly low levels in parasite-exposed cells. Investigation of the KEGG PATHWAY database demonstrates that ezrin has important roles in various pathways, particularly, regulation of actin cytoskeleton, which has implications to malaria, as shown in Figure 4c. Ezrin exerts its biological functions through protein-protein interactions and its active form is regulated by Rho kinase/ROCK2, which directly interacts with membrane proteins [31,72]. Ezrin binds to adhesion-related proteins with single transmembrane domains such as ICAM-1, CD44 and CD43 through their cytoplasmic tails to modulate cell morphology [61,73]. Cytoadhesion molecules such as ICAM1 contribute to cytoadhesive phenotype/rosetting and high intensity of rosetting is found in anaemia cases with *P. vivax* infection [74]. Both ICAM-1 and VCAM-1, soluble adhesion molecules, are detected at high levels in serum from falciparum patients with severe malaria [75]. This study also found abnormal erythroid cell aggregation in culture of gECs exposed to IE lysates (Additional file 2). Taken together, this suggests that during interaction of *P. vivax* proteins with adhesion molecules on erythroid cells, ezrin regulates cell adhesion by connecting membrane adhesion receptors to the actin-based cytoskeleton. This erythroid cell adhesion may contribute to down regulation of erythroid cell production leading to the development of anaemia. For other adhesion molecules, integrin alpha L chain (ITGAL) and integrin α2β1 has been found a higher probability to involve in severe thrombocytopenia in vivax malaria [76]. The expression of molecules sharing an epitope with human ITGB2/LFA-1 integrin, or CD18 leukocyte integrin on the *Plasmodium falciparum*-parasitized erythrocyte surface could be involved in the pathogenesis of severe disease [77]. However, further studies are need to explore the association of these intercellular adhesion molecules with ezrin in vivax malaria.

In addition, ERM proteins are members of the band 4.1 superfamily, FERM (four-point one, ezrin, redixin, moesin) [78-80]. Phosphorylation of erythrocyte protein 4.1 is involved with the modification of the host erythrocyte membrane by *P. falciparum* [81]. Moreover, tyrosine phosphorylation of band 3, band 4.2, catalase and actin in *P. falciparum*-infected erythrocytes are predicted to be part of the regulatory mechanism to modify the erythrocyte membrane [82]. This study found low levels of alpha actinin-1 in gECs in the presence of IE. The subsequent reassembly of actin structure in response to parasite suppression is currently being investigated. This poorly understood aspect of ezrin function suggests that parasites inhibit the ezrin protein allowing the assembly of complex cellular structures in erythroid cells leading to dyserythropoiesis, inhibition of erythroid cell growth and division resulting in the accumulation of cells in a resting G0 phase. This study demonstrates that *vivax* parasites suppress development of erythroid progenitor cells through a mechanism that includes decreased ezrin phosphorylation. *Plasmodium vivax* is able to enter bone marrow, as previously reported [17,20,57,83], and parasites or its products bind to erythroid progenitor cells, resulting in decreased ezrin phosphorylation, leading to suppression of erythroid development, and ultimately anaemia. This is the first analysis suggesting that ezrin contributes to the suppression of erythroid cell growth by *P. vivax*. Further investigation of this mechanism should help to better understand pathogenesis of anaemia in acute or chronic *P. vivax* infection.

## Conclusions

This analysis demonstrates that the pathogenesis of anaemia in vivax malaria is mediated by parasite suppression of human erythroid cell growth and division. Inactivation of the ezrin protein, leading to ineffective erythropoiesis and dyserythropoiesis, appears to be a key event resulting in the development of severe anaemia. The understanding of the pathogenesis of anaemia in vivax malaria should help in the development of therapeutic strategies to treat severe anaemia malaria.

## Additional files

Additional file 1:
**Intensity ratios of ezrin and phospho-ezrin Thr567 from gECs exposed to IE/UE relative to signals from gECs in medium control.** The intensity of ezrin and phospho-ezrin Thr567, from 1,000 gECs cells in each condition, exposed to IE/UE or in media were determined using ImageJ software (http://imagej.nih.gov). Intensity ratios were calculated using the mean intensity of IE/UE-exposed gECs normalized to the mean intensity from gECs in media. Additional file 2:
**Erythroid cell aggregation in culture of gECs exposed to** ***P. vivax*** **.** Erythroid cells, 5-day old, were cultured with IE/UE lysates at a ratio of 1:10 (gEC:IE/UE) for 24, 48 and 72 h. Cell aggregation was observed under inverted microscope with 200x magnification.

## Abbreviations

(gECs): Growing erythroid cells
(IE): Infected erythrocyte
(UE): Uninfected erythrocyte
(ERM): Ezrin ezrin radixin and moesin
(HSCs): Haematopoietic stem cells
(Thr567): Threonine 567
(LC-MS): Liquid chromatography mass spectrometry
