# Supplementary figures and images for: Plasmodium vivax inhibits erythroid cell growth through altered phosphorylation of the cytoskeletal protein ezrin
Source: Malar J. 2015 Mar 31;14:138. doi: 10.1186/s12936-015-0648-9 (PMC4392472; doi:10.1186/s12936-015-0648-9)

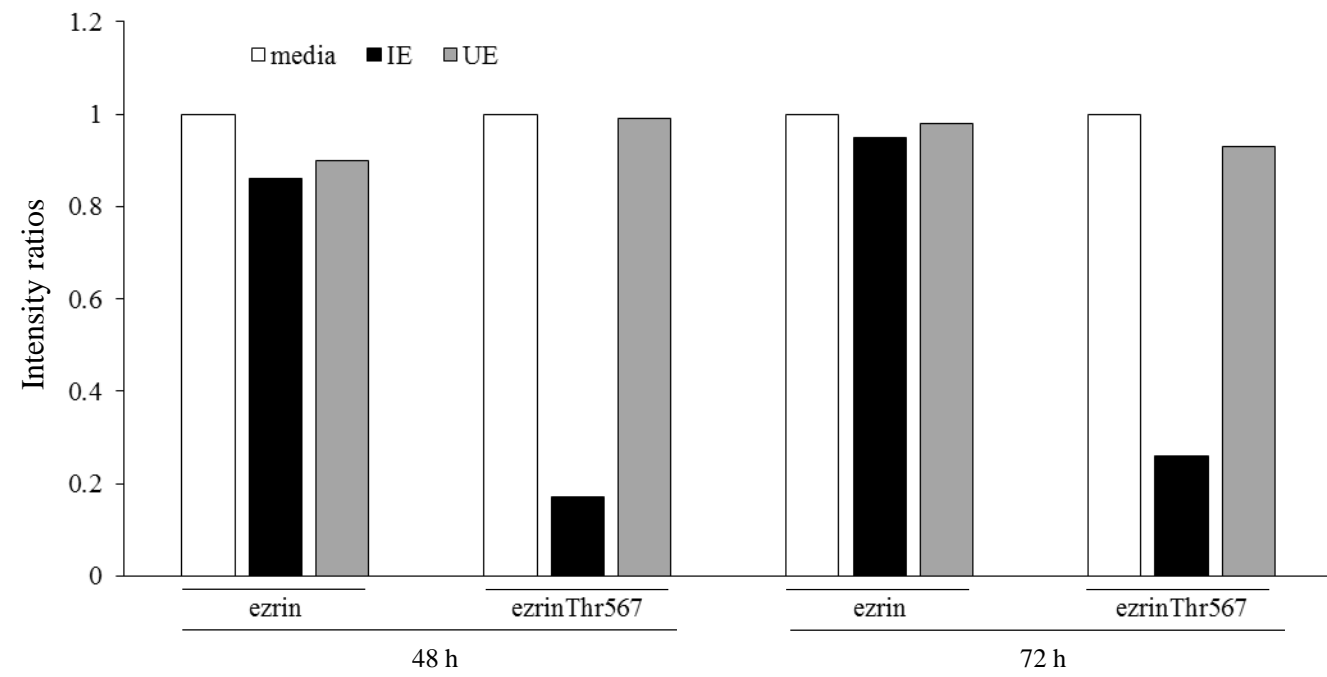

Supplement: Additional file 1: — Intensity ratios of ezrin and phospho-ezrin Thr567 from gECs exposed to IE/UE relative to signals from gECs in medium control. The intensity of ezrin and phospho-ezrin Thr567, from 1,000 gECs cells in each condition, exposed to IE/UE or in media were determined using ImageJ software (http://imagej.nih.gov). Intensity ratios were calculated using the mean intensity of IE/UE-exposed gECs normalized to the mean intensity from gECs in media. [file 12936_2015_648_MOESM1_ESM.pdf]

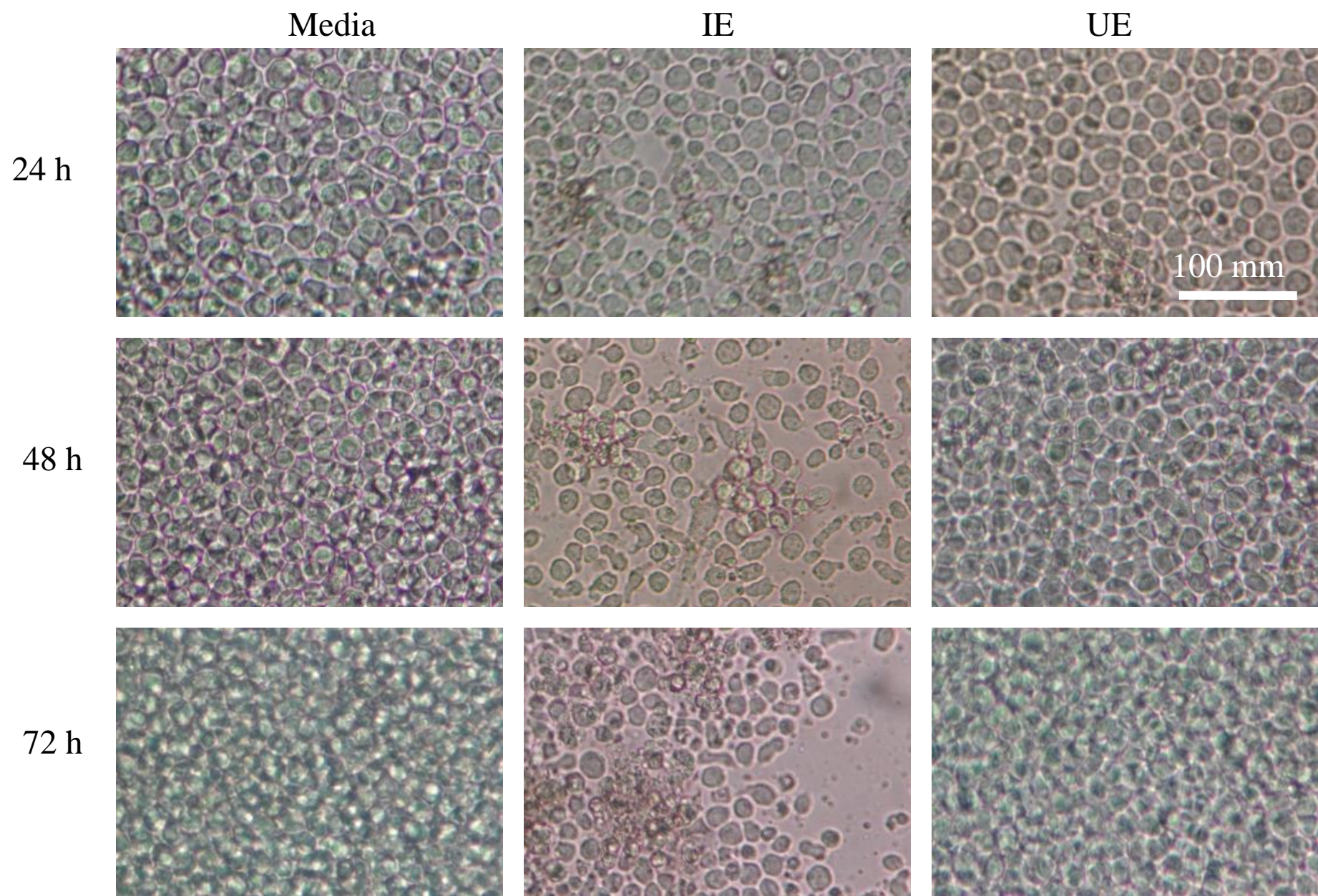

Supplement: Additional file 2: — Erythroid cell aggregation in culture of gECs exposed to P. vivax . Erythroid cells, 5-day old, were cultured with IE/UE lysates at a ratio of 1:10 (gEC:IE/UE) for 24, 48 and 72 h. Cell aggregation was observed under inverted microscope with 200x magnification. [file 12936_2015_648_MOESM2_ESM.pdf]
